# Partial Pericardial Agenesis

**DOI:** 10.14797/mdcvj.1232

**Published:** 2023-05-16

**Authors:** Akila Bersali, Faisal Nabi

**Affiliations:** 1 Houston Methodist Academic Institute, Houston, Texas, US; 2 Houston Methodist DeBakey Heart & Vascular Center, Houston Methodist Hospital, Houston, Texas, US

**Keywords:** congenital absence of pericardium, cardiac MRI

## Abstract

Congenital absence of the pericardium is a rare anomaly, affecting the left pericardium (86%) more than the right, with male predilection distribution (3:1). In the majority of cases, the condition is asymptomatic. We describe a case of a 55-year-old female with a history of chronic hypercapnic respiratory failure secondary to restrictive lung disease who was referred to cardiovascular magnetic resonance (CMR) lab for shunt evaluation based on right ventricular pressure overload and paradoxical septal motion.

Congenital absence of the pericardium is a rare anomaly, affecting the left pericardium (86%) more than the right, with male predilection distribution (3:1). In the majority of cases, the condition is asymptomatic.^1^,^2^,^3^

Cardiac magnetic resonance imaging (MRI) reveals the following features:

1. Nonvisualization of the pericardium—low signal on T2
2. Levoposition of the heart
3. Presence of lung tissue:
  - between the heart and other thoracic structures,
  - between the main pulmonary artery and aorta, which is considered a very specific sign, and
  - between the diaphragm and the inferior wall of the heart.

Additionally, nonvisualization of the left-sided pericardium is also a feature of transthoracic echocardiography on both subcostal views.

We report a case of a 55-year-old female with a history of chronic hypercapnic respiratory failure secondary to restrictive lung disease. She was referred to our cardiac magnetic resonance (CMR) lab for shunt evaluation based on right ventricular pressure overload and paradoxical septal motion. CMR revealed interposition of lung tissue (Figure 1) (A) between the main pulmonary artery and aorta on the axial localizer view, and (B, Video 1) between the diaphragm and inferior LV wall on a coronal T2 weighted sequence. A loss of direct visualization of the left-sided pericardium was noted on both (C) the half fourier single-shot turbo spin-echo (HASTE) sequence, and (D, Video 2) the subcostal view on transthoracic echocardiography.^4^,^5^,^6^ On top of that, we can highlight the leftward position of the apex on the HASTE sequence.

**Figure 1:**
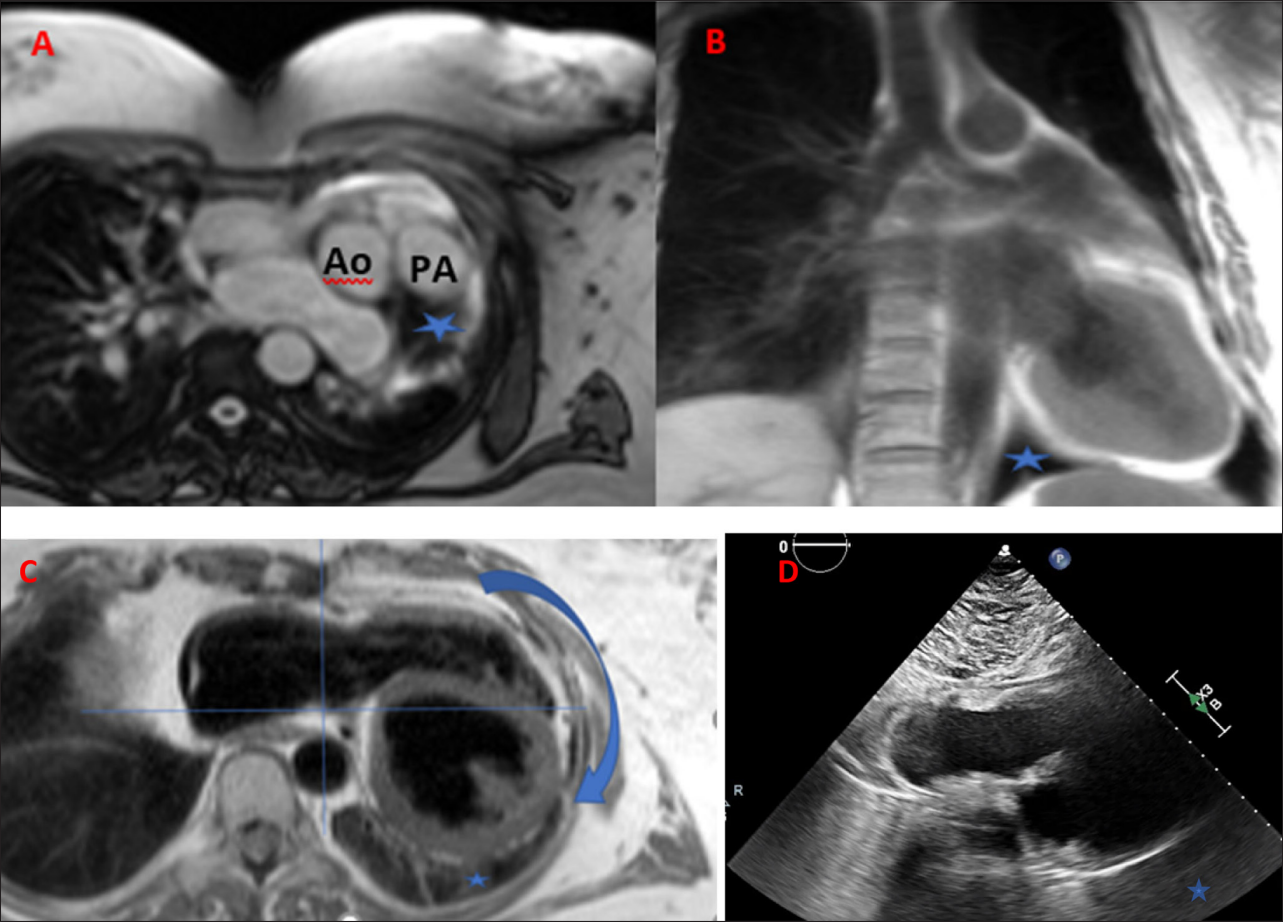
Cardiac magnetic resonance imaging of **(A)** axial localizer view and interposition of lung tissue between the main pulmonary artery and aorta (blue star), and **(B)** between the diaphragm and inferior left ventricle wall on coronal T2 weighted sequence. **(C)** HASTE (half fourier single-shot turbo spin-echo) sequence shows leftward position of the apex and loss of direct visualization of the left-sided pericardium as well as on the **(D)** subcostal transthoracic echocardiogram view. Ao: aorta; PA: pulmonary artery

**Video 1:** Presence of lung tissue between the diaphragm and the inferior left ventricle wall on coronal cine steady-state free precession sequence; see also at https://youtu.be/X3DEOwWOwNI.

**Video 2:** Nonvisualization of the left-sided pericardium; see also at https://youtu.be/fXcX_OSK8io.
